# Hypothermia from a two‐component mixture comprising Amoxicillin and Sulbactam

**DOI:** 10.1002/ccr3.2829

**Published:** 2020-04-10

**Authors:** Tung Anh Dinh Duong, Ngoc Anh Hoang, Linh Doan Thi, Dung Bui Thi, Huong Pham Thi, Sang Nguyen Ngoc, Chien Bui Van, Thuc Dinh Van

**Affiliations:** ^1^ Pediatrics Department Haiphong University of Medicine and Pharmacy Haiphong Vietnam; ^2^ Respiratory Department Haiphong Children's Hospital Haiphong Vietnam

**Keywords:** amoxicillin, hypothermia, pneumonia, sulbactam

## Abstract

Hypothermia might be an adverse effect of Amoxicillin and/or Sulbactam, and clinicians should be aware of this effect. Further clinical and laboratory investigations are also needed to confirm and clarify the underlying mechanism of this side effect.

## Graphical abstract

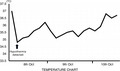

## 1 INTRODUCTION

The ability to maintain the resting body temperature around 37°C is a key feature of human survival.^1^ It has been reported that physiological impairments and fatality might occur when a deviation of ±3.5°C from this resting core temperature.^2^ Normal thermoregulation might be interfered with many drugs including antibiotics, resulting in hypothermia which is defined as a body temperature below 35°C.^3^, ^4^ Here, we report a case of hypothermia following a treatment with a two‐component mixture comprising Amoxicillin and Sulbactam.

## 2 CASE PRESENTATION

**Figure 1:**
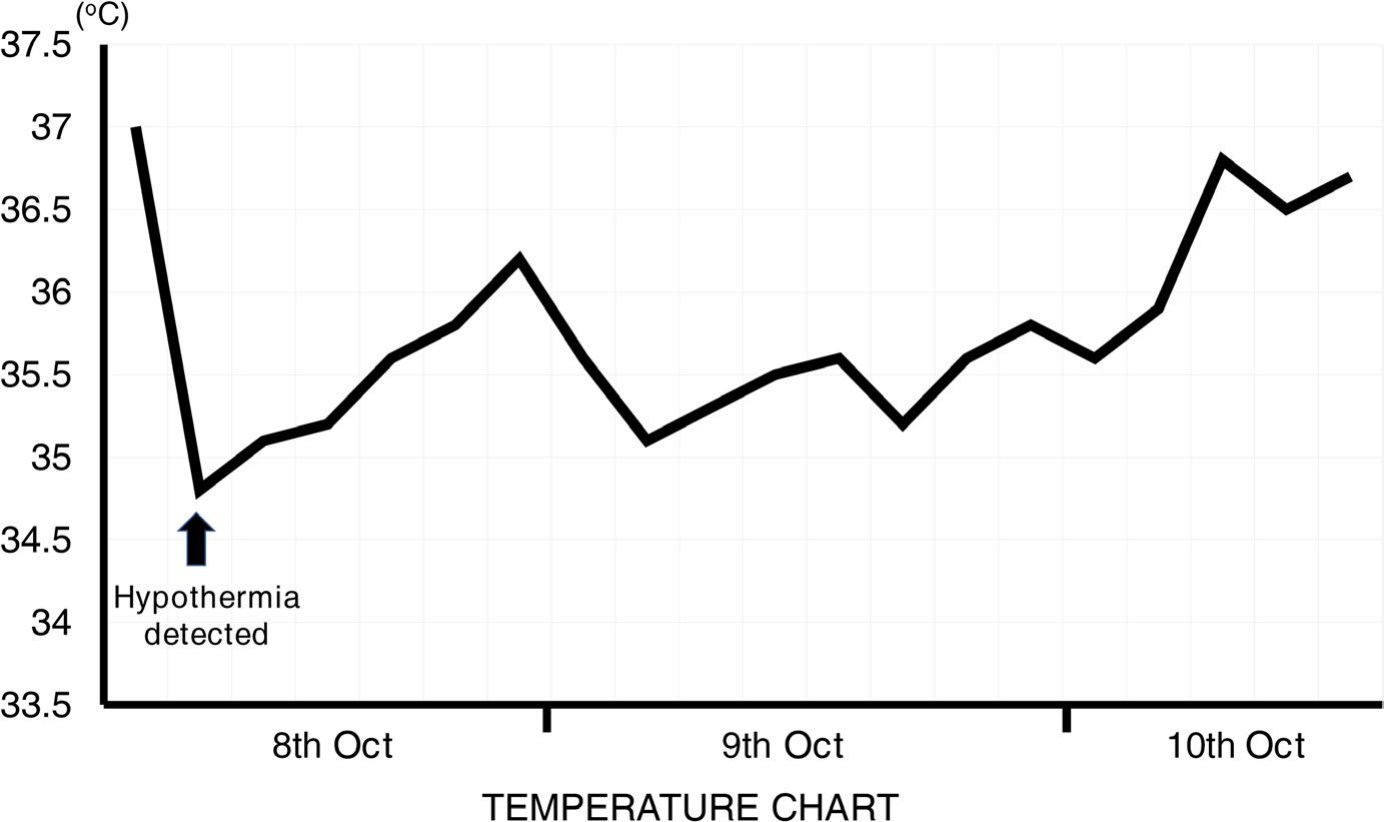
Hypothermia from a mixture of Amoxicillin and Sulbactam. Hypothermia (at 34.8°C) was detected at 30 min after the injection of a mixture of Amoxicillin sodium (100 mg/kg/d) and Sulbactam sodium (50 mg/kg/d) on a 2‐year‐old boy with pneumonia. His temperature was assessed every three hours. His temperature recovered to 36.6°C at three days after the discontinuation of treatment

A 2‐year‐old boy weighing 10.6 kg without no known allergies was hospitalized due to cough. At the time of the hospital admission, his physical examinations showed that he was fully conscious. He also had fever (38.5°C), tachypnea (42 rpm) without hypoxia, and bilateral crackles. The chest radiography was consistent with pneumonia. The white blood cell count revealed a leukocytosis [WBC count: 24.2 × 10^9^ cells/L, NEU count: 19.6 × 10^9^ cells/L (81%), and LYM count: 4.1 × 10^9^ cells/L (17%)]. Level of C‐reactive protein was found clearly elevated (114.41 mg/L). Furthermore, *QuantiFERON*‐TB Gold test was negative. He was diagnosed with pneumonia without any relevant comorbidity and treated by Amoxicillin sodium (100 mg/kg/d) and Sulbactam sodium (50 mg/kg/d), IV twice per day. Unfortunately, sputum culture could not identify any causative pathogen. After the first three days of antibiotic therapy, his pneumonia had much ameliorated: no fever, less cough, and much fewer crepitations. Unexpectedly, 30 mins after the ninth antibiotic injection in the morning of the fourth day of treatment, his skin became cold and pale and he involuntarily shivered. The temporal rectal temperature was at 34.8°C (Figure 1). The patient was in good consciousness with a normal blood pressure. We did not detect any sign of tachycardia or tachypnea. The treatment by Amoxicillin and Sulbactam was withdrawn. Thirty mins after being covered with a warm blanket and given warm milk, his temperature raised up to 35.1°C. We assessed his temperature every three hours. His rectal temperature recovered to 36.6°C at 3 d after the discontinuation of treatment (Figure 1). One day later, the patient was discharged. It is important to remark that the boy had been administered solely with Amoxicillin twice before to treat a pharyngitis (two months ago) and a bronchitis (a month ago) without any recorded hypothermia.

## 3 DISCUSSIONS

Even though our patient presented a mild hypothermia,^4^ it is worthy to remark that this case of hypothermia was detected at an early time just after the given injection of the mixture of Amoxicillin and Sulbactam, raising the possibility that the antibiotic injection might be at the origin of this hypothermia and that his body temperature might be severely reduced if detected at a later time. As the boy was treated with Amoxicillin twice before without any remarkable side effect, we suspect that his hypothermia could be due to solely Sulbactam or to the drug interaction between Amoxicillin and Sulbactam. Other studies have revealed different side effects of Amoxicillin, such as diarrhea, nausea, skin rash, vulvovaginal irritation, anal irritation,^5^ or crystalluria.^6^ Previously, the treatment using Sulbactam associated with ampicillin has been reported to induce several side effects, such as mild diarrhea, nausea, headache, oral or vaginal candidiasis, and occasionally generalized rash, urticaria^7^.

Previously, other researches have also described different cases of hypothermia from antibiotic treatments such as erythromycin, azithromycin, or penicillin.^8^, ^9^, ^10^ Consistently, hypothermia was also detected short time after the drug administration in these studies and it disappeared few days after the cessation of the antibiotic therapy.

## 4 CONCLUSION

Because there is no reported case of hypothermia due to Amoxicillin or Sulbactam treatment, to our knowledge, this case report might be the first alert on a newly remarkable side effect of the mixture of Amoxicillin and Sulbactam. Hypothermia might be an adverse effect of Amoxicillin and/or Sulbactam, and clinicians should be aware of this effect. Further clinical and laboratory investigations are also needed to confirm and clarify the underlying mechanism of this side effect.

## CONFLICT OF INTEREST

None declared.

## AUTHOR CONTRIBUTIONS

TADD and NAH contributed equally to this manuscript. TADD, NAH, and TDV designed research. HPT, SNN, and CBV contributed to the observation of the patients during the treatment. LDT and DBT analyzed patient's temperature. TADD, NAH, and TDV wrote the report.

## References

[1] Lim CL , Byrne C , Lee JK . Human thermoregulation and measurement of body temperature in exercise and clinical settings. Ann Acad Med Singapore. 2008;37(4):347‐353. 18461221

[2] Moran DS , Mendal L . Core temperature measurement: methods and current insights. Sports Med. 2002;32(14):879‐885. 12427049 10.2165/00007256-200232140-00001

[3] Donati M , Monaco L , Melis M , et al. Ibuprofen‐associated hypothermia in children: analysis of the Italian spontaneous reporting database. Eur J Clin Pharmacol. 2016;72(10):1239‐1243. 27417946 10.1007/s00228-016-2088-z

[4] Paal P , Gordon L , Strapazzon G , et al. Accidental hypothermia‐an update : The content of this review is endorsed by the International Commission for Mountain Emergency Medicine (ICAR MEDCOM). Scand J Trauma Resusc Emerg Med. 2016;24(1):111. 27633781 10.1186/s13049-016-0303-7 PMC5025630

[5] Neringer R , Stromberg A . A comparison of the side‐effects of amoxycillin and pivampicillin. Scand J Infect Dis. 1980;12(2):133‐135. 6990474 10.3109/inf.1980.12.issue-2.11

[6] Hentzien M , Lambert D , Limelette A , et al. Macroscopic amoxicillin crystalluria. Lancet. 2015;385(9984):2296 10.1016/S0140-6736(14)62001-8. 25680270

[7] Hassel B . Hypothermia from erythromycin. Ann Intern Med. 1991;115(1):69‐70. 10.7326/0003-4819-115-1-69_2 2048867

[8] Hassel B . Acute hypothermia due to penicillin. BMJ. 1992;304(6831):882. 10.1136/bmj.304.6831.882-b PMC1882814 1392751

[9] Kavukcu S , Uguz A , Aydin A . Hypothermia from azithromycin. J Toxicol Clin Toxicol. 1997;35(2):225‐226. 9120898 10.3109/15563659709001202

[10] Shorr RIH , Rawls N . Drugs for the Geriatric Patient. Saunders. 2007;995.

